# The Effectiveness of the Psychological Intervention in Amateur Male Marathon Runners

**DOI:** 10.3389/fpsyg.2021.605130

**Published:** 2021-03-24

**Authors:** Jose C. Jaenes, Dominika Wilczyńska, David Alarcón, Rafael Peñaloza, Arturo Casado, Manuel Trujillo

**Affiliations:** ^1^Department Social Anthropology, Basic Psychology and Public Health, University Pablo de Olavide, Seville, Spain; ^2^Department of Social Science, Gdansk University of Physical Education and Sport, Gdańsk, Poland; ^3^Faculty of Higher Studies Zaragoza, Psychology Career, National Autonomous University of Mexico, Mexico City, Mexico; ^4^Centre for Sport Studies, Rey Juan Carlos University, Madrid, Spain; ^5^School of Medicine, New York University, New York City, NY, United States

**Keywords:** psychological preparation, track and field athletes, performance, negative thoughts, anxiety

## Abstract

**Background:** The Marathon runners must have the proper technical preparation to reach excellence and to achieve adequate psychological preparation for the race. Against this background, the current study aims to describe the implementation results of a cognitive-behavioral intervention based on psychological skills training for marathon runners.

**Methods:** Fourteen amateur male marathoners with an average age of 30 (*SD* = 5.75) were trained with various emotional and cognitive control techniques to enhance their performance in competition. Various psychological variables, related to the subjects level of perceived stress, and to qualitative characteristics of their thoughts were measured before and after the target marathon race. Results were analyzed through non-parametric tests for two related samples. The Cohen's *d* effect size for single-group pretest-posttest repeated measures were also performed.

**Results:** Statistical analysis reveals that, controlling for age and running experience, the intervention decreased significantly the level of perceived stress and the occurrence of negative thoughts before the race, during, and after the race.

**Conclusion:** Training in cognitive control and relaxation techniques, as part of the psychological skills training could determine the quality of performance of marathon runners.

## Introduction

Running long distances, specifically a 42,195-meters race, is a physically and psychologically demanding task. While elite runners can complete it in just over 2 h, recreational runners rarely finish the race in under 3 h, and many can take up to five or more hours. Sport psychology researchers became interested in this demanding distance sport in the 1970s and 80s, investigating different aspects of the athletes involved, such as commitment (Carmack and Martens, 1979; Joseph and Robbins, 1981), runners psychosocial characteristics (Gontang et al., 1977; Freischlag, 1981; Summers et al., 1983), the presence of running addiction (Little, 1976; Sachs, 1982; Sacks, 1982; Glasser, 1985; Morgan et al., 1987; Jaenes, 1994; Szabo et al., 1997), or the use of a marathon race as an adjuvant in psychotherapy in order to attenuate the impact of selected psychiatric, psychosomatic, and somatic syndromes (Solomon and Bumpus, 1978).

Previous research focuses on marathoner's psychological characteristics such as their level of anxiety (Masters and Lambert, 1989), the possible relationship between cognitive and somatic anxiety with self-confidence (Jaenes, 1994; Jaenes and Caracuel, 2016), and the socio-demographic predictors of anxiety level among runners (Ruiz-Juan and Zarauz, 2014; Ruiz-Juan et al., 2016). Studies regarding cognitive and somatic anxiety, have reported that marathoners are, in many cases, individualists, and as such, they perform better when their level of cognitive anxiety is medium to high (Raglin and Hanin, 2000; Jaenes and Caracuel, 2016). Other authors observed that the most successful runners showed minimal rates of undesirable moods and some adverse emotional characteristics such as anger, fatigue, depression, and confusion, while reporting higher rates of “vigor” (Morgan and Pollock, 1977; Okwumabua et al., 1983; Morgan et al., 1987). Elite marathoners are also characterized by intrinsic motivation, and by a very high level of motivation to achieve goals (Morgan et al., 1987; Foster and Walker, 2005). Other authors have reported these athletes' high levels of hardiness (Jaenes et al., 2009), and the capacity to experience “flow state” while achieving their best results (Fernández Macías et al., 2015). Studies which compare long-distance runners of different performance levels show that higher performance level athletes are significantly more focused on their specific training activities and tended to have more fun performing these activities, than lower-level runners (Casado et al., 2014; Casado and Ruiz-Pérez, 2017).

Running addiction, and its predictors among long-distance runners, has also been investigated (Ruiz-Juan and Zarauz, 2012; Ruiz-Juan et al., 2019), together with running addiction consequences such as the presence of eating disorders (Yates, 1987, 1991; Hausenblas and Carron, 1999; Davis and Strachan, 2001; Yates et al., 2010). Some authors also tested the impact on runners of different intervention methods, such as the cognitive association-dissociation technique (Okwumabua et al., 1983; Schomer, 1986, 1987; Takai, 1996, 1998; Wulf, 2013; Brick et al., 2014).

Research results have consistently supported the idea that psychological skills training (PST) can increase an athletes' sport enjoyment and performance (Martin et al., 2012). The importance of PST in the development of athletic performance is widely recognized (Birrer and Morgan, 2010), even in long-distance runs (Thelwell and Greenlees, 2003). PST better demonstrates its efficacy when used in participants in high-intensity sports, as it facilitates the interpretation of cognitive and somatic sensations, helps the athlete to manage pain, and to use associative attentional techniques (Birrer and Morgan, 2010). PST techniques also help in managing the intensity and directional dimensions of competitive anxiety when relaxation techniques are added (Wadey and Hanton, 2008). According to McCormick et al. (2015), in a systematic literature review, PST consistently improves endurance performance, and thus benefits endurance athletes. There are studies focused on the use of cognitive strategies (Schomer, 1986, 1987), to reduce anxiety and increase self-confidence (Jaenes and Caracuel, 2016), and on the utility of Riera's skill training (Riera, 2005, 2010). Based on the cumulative effect of this research on professional athletes, the current study investigates the impact of cognitive-behavioral intervention, based on psychological skills training (PST) on amateur athletes who practice marathons, a population far larger and less studied than that of elite runners.

The PST program appeared to foster relaxation, concentration, and refocusing skills in young gymnasts (Fournier et al., 2005). Vesković et al. (2019) studied the effects and effectiveness of a psychological skills training program on the optimization of anxiety and self-confidence in a group of karate athletes. Fletcher and Hanton (2001) demonstrated the efficacy of the PST program in swimmers using relaxation strategies to reduce and interpret their intensity levels. Similar results were found about the effect of two different psychological methods of skills training—self-talk and goal setting—on youth swimmers' swimming performance (Meggs and Chen, 2019). The 5-week psychological skills intervention program effectively improved youth swimming performance (Meggs and Chen, 2019). Schuler and Langens (2007) investigated the buffering effects of self-verbalization in a marathon race in two different studies, in the first study they found that a major psychological crisis occurs around kilometer 30, a crisis characterized by a strong urge to disengage from the goal and thoughts about benefits and costs, and that this crisis had negative effects on race performance. In a second study, the use of self-verbalizations was experimentally induced. The results confirmed the hypothesis that self-talk effectively buffer against the psychological crisis's adverse effects on race performance.

Despite the demonstrated effectiveness of PST interventions in the athletes' population, resistance to the use of PST has been reported often, and it can be related to the presence of barrier such as gender (Gnacinski et al., 2017). This study confirms that female athletes are more receptive and open to sport psychology consultation than male athletes. Female athletes are more willing to work with a sport psychologist, are less likely to stigmatize services, and have more trust in the usefulness of psychological skills training consultations than male athletes. Research on athletes' expectations has also found female athletes to be more committed (e.g., higher levels of self-responsibility, openness, and motivation) to the sport psychology consulting process than their males counterparts (Martin, 2005; Wrisberg et al., 2009; Martin et al., 2012). Therefore, this study will also assess the influence of a 7-week intervention, based on selected PST techniques, specifically on male recreational athletes. This PST intervention has been designed to help marathon runners to cope with the psychological demands of the race by providing them with skills and techniques to deal with their emotional challenges. We hypothesize that the PST intervention will decrease participants' experienced stress and negative thoughts about the race.

## Methods

### Participants

Fourteen male amateur marathon runners with an average age of 30 (*SD* = 5.75) participated in the current study. All the participants were recruited at the Andalusian Center of Sport Medicine (CAMD) where they were participating voluntarily in psychological training with a sport psychologist. The majority of subjects had finished a minimum of three marathons, and an average of 16 years of experience (*SD* = 5.11). All the subjects were registered to participate in the 2017 Seville International Marathon held in Spain.

### Materials

For the intervention, a recording with the Progressive Jacobson Relaxation Technique adapted to long-distance runners was used. This kind of technique is commonly practiced by athletes while participating in psychological training in the Psychology Services at Andalusian Center of Sport Medicine. The relaxation practice was recorded on a Redmi Note 6 Pro Telephone Terminal, during the group session (lasting 12 min and 16 s), which was sent to the terminals of all participants. To assess the athletes' mental state, a self-report measure was created for purpose of the present study, most of the items were based on the validated Spanish version of the Competitive State Anxiety Inventory 2 (CSAI-2R) questionnaire (Fernández et al., 2007), and the others items were created for purpose of the present study, see Table 1. The instrument consisted of 8 statements: two statements described probable negative thoughts before the race, the next four described possible negative thoughts during the race, and the last two statements characterized the self - doubts and mental load, the participants could experience after the race. All items were answered on a 5-point Likert scale ranging from 1 (totally disagree) to 5 (totally agree).

**Table 1:** Athletes' mental state self-reported questionnaire.

1. Before the marathon, I am concerned about the race.
2. Before the marathon, I am concerned I may not do as well in this race as I could.
3. During the marathon, I am worried about reaching my goal.
4. During the marathon, I am concerned about shocking under pressure.
5. During the marathon, I am concerned I will not be able to concentrate.
6. During the marathon, I am concerned about performing poorly.
7. After the marathon, I am concerned about wasting more mental energy that was necessary.
8. After the marathon, I have self-doubts about that I had performed as I should.

*Items were answered on a Likert scale ranging from 1 (totally disagree) to 5 (totally agree)*.

### Procedure

Previous studies reported that the Psychological Skill Training (PST) technique was one of the most effective psychological interventions in sports (Neil et al., 2006; McCormick et al., 2015). The intervention program was based on the PST principles (Weinberg and Gould, 2014), and the sport classifications and interactions of Riera (2005, 2010). The cognitive-behavioral techniques employed in the PST program were selected according to the psychologists' experience training marathon runners, and by the recent literature about its efficacy. The following cognitive-behavioral techniques were employed in the PST program:

Cognitive Association technique: running focus on the pace, running pack, breathing, scanning muscular sensations, self-testing and monitory their body, making mind maps, underlying key words, self-positive messages, etc. For instance, when the attention was focused on the body, physical sensations, pain, the focus is internal.

Cognitive dissociation technique: when the attention was focused on anything than other and internal sensations, is dissociation (Master and Ogles, 1998). For instance, thinking on the family, and friends, other races, observing public attitudes during the race, etc. Rodríguez et al. (2017) has studied the influence of association and dissociation cognitive strategies on different physiological variables, in a group of experienced long distance runners in a controlled condition in laboratory, and how both strategies affect performance at different running pace.

Thought-stopping technique: is a cognitive technique involving mental or behavioral aspects (Zinsser et al., 2006). The aims are to disrupt negative thinking patterns, removing, replacing, and redirect problematic thoughts to something that helps relieve distress. Otherwise, focusing on the relevant stimulus while running and disrupt unwanted thoughts. It is an active process of suppressing self-doubts, negative ideas along 42 kilometers, through the personalized self-talk to enhanced performance (Magnusson and van Roon, 2013).

Jacobson's relaxation or progressive relaxation technique: is a type of therapy that focuses on tightening and relaxing specific muscle groups, in the case of marathoners consists of three times -three times right arm, left arm, and then three-time right leg and three the left leg in sequence, following by visualization of a relaxing situation. By focussing on specific muscle groups and tensing-relaxing them, athletes can become more aware of their body and physical sensations (Chang et al., 2020).

Coordination skills technique: coordination skills during a race are important behaviors that a runner should train for a marathon race, they included a group of instrumental skills: open a bottle without coughing, keeping pace with the race, drink isotonic gels; and tactical skills: running ahead, running in the middle of the group, slowing, or speeding up a pace (Riera, 2005, 2010; Jaenes and Caracuel, 2016).

Following Jaenes and Caracuel (2016), the intervention focused on the leading Marathon runners psychological training skills. It was carried out over 7 weeks before the marathon, with a weekly session lasting between 60 and 90 min each. The first two sessions were implemented in groups of seven runners, and the remaining training sessions were performed individually. Table 2, below, shows the schedule of the sessions and their content.

**Table 2:** Schedule of the sessions of the psychological intervention program in marathon runners.

**Week**	**Modality**	**Content**
1	Group	Presentation and demonstration of the psychological training and teaching behavioral-cognitive techniques to the participants as well as base-line evaluation.
2	Group	Teaching cognitive behavioral techniques (cont.).
3	Individual	Adjustment session (techniques) and individual assessment.
4	Individual	Adjustment session (techniques) and individual assessment.
5	Individual	Adjustment session (techniques) and individual assessment.
6	Individual	Adjustment session (techniques) and individual assessment.
7	Individual	Race and evaluation of the effectiveness of the intervention.

The PST's sessions topics, as well as selected cognitive-behavioral techniques (such as Association-Dissociation Technique and the Pause of Thoughts Technique), were explained during the first meeting. Furthermore, the Jacobson Relaxation Technique, adapted to marathon runners, was requested to be practiced once each day. The participants were advised to practice these training techniques in three different situations: (1) at home three times per day; (2) when negative thoughts arose during training; and (3) after self-provoking the distracting thoughts during the actual training running.

The PST program had the following three sessions: First, Education phase: runners and psychologist make a self-presentation, psychologist introduced what is PST, and the potential benefits of psychological skills to cope with different situations before and during a marathon, based on their psychological needs refers to the sport psychologist in previous meetings. The program characteristics, components, and information about the cognitive techniques were provided, and the base-line runners' evaluation (pre) was collected with the mental scale. Second, Acquisition phase: A 7 weeks of specific individual training was carried out by the athletes supervised by a sport psychologist. This crash-training is considered enough training to run and compete in a marathon (Péronnet et al., 2001; Jaenes and Caracuel, 2016). During the 7 weeks of practice, see at Table 2, most of the practice was performed while athletes were running, except for the relaxation technique -one time every day at home-. The psychologist made a continuous adjustment of the training to adapt the PST program according to the athlete sensations and performance, but the modifications did not alter the PST program's main structure. Third, Evaluation phase: post evaluation with the mental scale was collected 48 h before the athletes competed at the marathon.

The PST's schedule and the content of 1 week are summarized in Table 3. In the subsequent sessions, individual adjustments were made to the different strategies and continued until the day of the race. For more information about the intervention, please contact the first author.

**Table 3:** Example of the weekly psychological training plan.

**Day**	**Training**	**Psychological Training**
Monday, Wednesday, and Friday	18 kms. Continuous race + stretching	The athlete must question himself/herself through self-dialog about the negative thoughts and doubts on the race, and apply the techniques to stop negative thougths in the race.He trains the coordination ability to be able to drink during the race.
Tuesday,	Warm-ups + (10 × 1.000, 1'15” recovery) meters at marathon pace + 3 kms. of jogging	Run 5 series in association, a cognitive attentional strategy (focusing on the sensations during the race), and 5 series in dissociation, another cognitive attentional strategy (ignoring the sensations during the race), and calculate the final time of each series. Write down about training effectiveness and feelings.
Thursday	Warm-ups + 2 × (10 × 500 meters, 45”−60” recovery) meters at marathon pace + 3 kms. of jogging	Run 5 series in association, a cognitive attentional strategy (focusing on the sensations during the race), and 5 series in dissociation, another cognitive attentional strategy (ignoring the sensations during the race), and calculate the final time of each series. Write down about training effectiveness and feelings.
Saturday	Warm-ups + (1 × 1.000, 2'15” recovery + 3 × 3,000, 3'15” recovery) marathon pace + 3 kms. of jogging	Practice the association strategy in the series (focused on the sensations during the race), calculate the total time of each series through his/her own sensations and without checking the chronometer
Sunday	2 h of continuous running (group training)	General practice for group racing tactics: running at the head, at the rear, at the middle, pulling and slowing down. Drinking and taking gels, running with the competition material to check the adaptation, provoking negative thoughts and cutting them out with self-dialogue training: association-dissociation.Evaluation of the week.

### Data Analysis

Non-parametric analyses were performed using the statistical program SPSS V. 25, to compare related samples and thus determine the intervention effectiveness evaluation. Following Morris and DeShon (2002), the Cohen's *d* effect size were reported for single-group pretest-posttest repeated measure correcting for the correlation; considering large effect sizes those >0.80. The internal consistency of the scale was evaluated by the Alpha's Crombach coefficient obtaining a value of α = 0.690, which is considered acceptable for scales with less than ten items (Loewenthal and Lewis, 2018).

### Ethical Approval

The study was approved by Andalusian Center of Sport Medicine (CAMD). The present study was conducted at the Sport Psychology Unit at CAMD, a public medical center under the direction of Andalusian Council of Education and Sport. Professionals and athletes are regulated under laws of strict confidentiality, and the CAMD's Ethical Committee approved the intervention. CAMD's objective is to care for the athlete's health in a holistic fashion. Ethical principles did not permit us to select a control group between athletes if they had required psychological attention at the Center.

## Results

Participants' age ranged from 21 to 40 (Mean = 30, *SD* = 5.76), they had a minimum of 9 years of running experience and a maximum of 28 years (Mean = 16.86, *SD* = 5.11). They had finished between one and eight marathons (Mean = 3.93, *SD* = 1.81). None of the results of the psychological measures used were significantly correlated with athlete's age, years of experience, or number of marathons finished.

**Figure 1:**
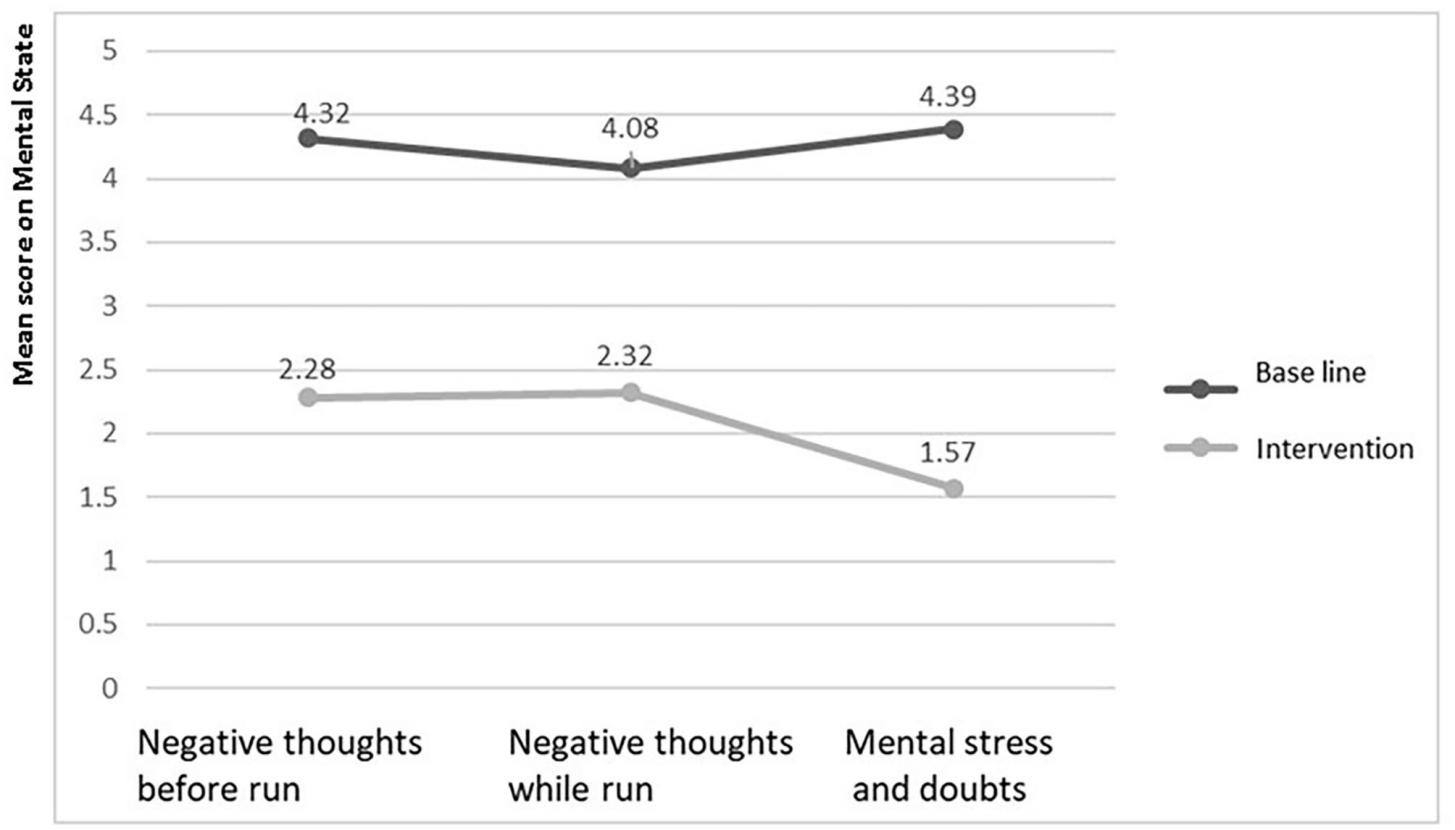
Comparison graph of the means for mental state measures in the basline and after the intervention.

Significant decreases in negative thoughts in all conditions (before, during, and after the race), as well as significant reductions in the experience of mental stress and self-doubts were observed after the treatment. Details about the efficacy of the intervention are presented in the figure below (Figure 1).

We tested that the assumption of independence of measurements for the before the race negative thoughts was met (*r* = −0.058, *p* > 0.05); and we found a *Z* value equals to −3.316 (*p* < 0.05), Cohen's *d* = 2.385. These results permit us to affirm that our sample marathoners, after the intervention, presented a significant decrease in negative thoughts prior to the race. The negative thoughts that were present during the race also showed independence between them (*r* = 0.204, *p* > 0.05). Their post-treatment decline was also significant (*Z* = −3,305, *p* < 0.05; Cohen's *d* = 3.051). Finally, statistically significant values were found regarding the perception of reduced mental load, and reduced self-doubts (*Z* = −3,307, *p* < 0.05; Cohen's *d* = 2.731); and the independence between measurements was verified as well (*r* = 0.197, *p* > 0.05). These data show that amateur non-professional marathoners significantly decreased self-doubts, and the perceptions of mental burdens after they were treated with the experimental intervention.

## Discussions

The current study supports the hypothesis that psychological skill training (PST), based on cognitive-behavioral techniques and Jacobson Progressive Relaxation technique, significantly decreases the appearance of negative thoughts, mental stress and self-doubt, in participants before, during and after the marathon race. Moreover, the most significant decrease in unwanted thoughts was noticed before and during the race. This result likely positively modified the mental load and reduced self-doubts, a combination of effects, which can effectively improve performance in such demanding tasks as a marathon. Buman et al. (2008) findings on selected cognitive coping responses to the experience of, speaking colloquially, “hitting the wall” often reported by recreational marathoners, point toward the need to develop efficient interventions to help them overcome such a disturbing experience. There are available a wide range of coping strategies designed to overcome extreme physical challenges. Among the most efficient mental strategies, which marathoners report using, are physical (such as supplementation/hydration) and behavioral (such as emotion-focused coping, social support, and cognitive strategies such as mental reframing). Moreover, Boyer (2008) underlines that thoughts associated with flexible inward monitoring (bodily sensations), and outward focusing (people and landscape) could partially protect runners from the experience of utter exhaustion that they describe as “hitting the wall.”

Martín et al. (1995) suggest that the running economy of competitive long-distance runners could be dependent on the implementation of different selected cognitive techniques (positive self-talk and associate techniques) during the race. As some authors underline, “hitting the wall” could also be prevented, or at least decreased, with adequate pacing. Research findings show that runners can be classified as having experienced “hitting the wall” if they ran any 1-km segment 11% slower than the average for the remaining segments on the race or if they ran any 5-km segment 7.3% slower than the average for the remaining 5-km segments on the race (Doherty et al., 2020). This means that using a rational and optimal pace through the whole marathon, performance with accessible and well-rehearsed strategies to be used at critical moments of mental fatigue, could help runners avoid or overcome the experience of “hitting the wall.”

Sport psychology recommends a large number of interventions that can be helpful for endurance athletes, but the effectiveness of these interventions has been questioned, and further effectiveness research has been frequently required (Weinberg and Comar, 1994; Solberg et al., 2000; Devonport, 2006; Ponnusamy and Grove, 2014; Keilani et al., 2016; Brown and Fletcher, 2017). The current study results show that a well-defined intervention based on PST has a significant effect in enhancing marathoners' capacity to cope with stress and with negative demoralizing thoughts about the race. These results support the findings of Meijen et al. (2017), proposing that marathon runners benefit from brief psychological interventions such as mental skill training before, during and after the race. The current study suggests that PST interventions are just as useful in general, and have the added benefit, of helping athletes to deal with the anxiety about the race. Psychological preparation is often ignored, or inadequately employed, in amateur long-distance runners (Solorio and Hickey, 2015). However, it is worth emphasizing that studies of first-time marathon runners highly recommend a multi-modal mental skills training approach as complementary race preparation to the well-established technical and physical training (Carter et al., 2016). Our study supports the idea that amateur runners can achieve robust enhancement of their psychological skills with an intervention such as PST, which is relatively easy to learn and time-efficient.

The current study main limitation is the absence of a control group. It was considered unethical to select a control group between athletes if they had required psychological attention at the Sport Psychology Unit. But a waiting-list control group should be employed in futures studies with similar restrictions. For a waiting-list control group desing, athletes attending to an introductory section should be randomly assigned to the experimental group (athletes who receive the PST program) or a waiting-list group (athletes who received the PST program 8 weeks later). Nevertheless, the procedure of the study is similar to many pedagogical experiments in sport training, the aim of which is expanding selected competencies and abilities of people through the educational process without excluding any participants (Karaulova et al., 2018). This non-excluding intervention is particularly relevant when training high-performance athletes and using innovative forms of intervention (Malikov et al., 2019, 2020; Boichenko, 2020). Many such rigourously designed uncontrolled studies have made important constributions. Thelwell and Greenlees (2003) presented a study that examined the effect of mental skill training (PST) on competitive gymnasium triathlon performance. They evaluated the impact of a training package including goal setting, relaxation, imaginary, and self-talk on the performance of four subjects without a control group; their results pointed out that a PST package was effective in enhancing all participants' performance. The results of our study show the effectiveness of PST in marathon amateur athletes and may serve to expand knowledge about psychological interventions in amateur and professional athletes.

Given the large effect sizes that were found in this study, despite the small sample size and lack of a control group, our results support the effectiveness of PST. We used statistical control of variables such as age, running experience, and number of completed marathons, that highlight the PST intervention as the most likely factor driving the improved performance: the significant decrease in negative thoughts and mental stress. We are planning future replication studies with control groups as well as studies of alternative interventions. For instance, an active control group may be used to compare the efficacy of PST with another type of training intervention. Longitudinal time series designs with baseline observations should be used to control underlying trends and to analyze the short, medium, and long-term effect sizes of the intervention (Eccles et al., 2003; Portela et al., 2015).

The current study provides a PST framework that could help psychologists and trainers to enhance athletes' mental skills, with promising applications for amateur and professional runners. Furthermore, as non-control studies are widely used in meta-analysis of training effectiveness (Weston et al., 2014; Csapo and Alegre, 2015; Taylor et al., 2015; Hammami et al., 2018; Stamatis et al., 2020), our results may be used in future meta-analysis to estimate the effects of PST on athletes.

## Data Availability Statement

The raw data supporting the conclusions of this article will be made available by the authors, without undue reservation.

## Ethics Statement

The studies involving human participants were reviewed and approved by the study was approved by the Andalusian Center of Sports Medicine. The patients/participants provided their written informed consent to participate in this study.

## Author Contributions

JJ conceived the study, designed the training, collected the data, and wrote the manuscript. DW made relevant contributions to the theoretical framework, interpretation of the results and revision of the final version. DA analyzed the data, wrote the reviewed results and discussion and provided critical revisions on the manuscript. RP analyzed the data and the model and provided critical revisions on the final drafts. AC reviewed the training program, collected the data, and make critical revisions on the manuscript. MT provided a final critical edit, as well the English revision. All authors read and approved the final manuscript.

## Conflict of Interest

The authors declare that the research was conducted in the absence of any commercial or financial relationships that could be construed as a potential conflict of interest.
